# Thyroid Treatment of Psoriasis

**Published:** 1909-01-16

**Authors:** 


### THYROID TREATMENT OF PSORIASIS.

Psoriasis is a disease which very frequently gives way for the time being before the internal administration of arsenic and the external application of ointments containing coal-tar products or chrysarobin. The two points to which most particular attention needs to be directed for the successful treatment of the case are, first, that the dose of liquor arsenicalis should be increased by 1 minim weekly until as much as 10 minims or more are being taken three times a day; and, secondly, that the external application must be mild in proportion to the acuteness of the eruption, and strong only in chronic cases. Neglect to observe the latter rule sometimes results in a serious dermatitis supervening on the psoriasis.

There remain certain cases, however, in which the above routine treatment fails to cure even temporarily, and then one is only too glad of any fresh suggestions. One such is the administration of five grains of thyroid extract three times a day, as recommended by Dr. Byrom Bramwell in his Clinical Studies. In some cases, without any external medication, the oral exhibition of thyroid extract is followed by rapid disappearance of the rash. Dr. Bramwell gives it as his opinion that although it will not prevent relapses any better than any other form of treatment does, in obstinate cases of psoriasis thyroid extract should always be tried, the effects being carefully watched in case of the development of untoward symptoms which might prevent its being continued.

The results of the treatment, extending over a series of cases, may be summarised as follows: (1) that in some cases—the exception rather than the rule—brilliant and remarkable results are obtained, small doses curing the condition rapidly; (2) that in some cases, if the remedy is given persistently, improvement, and, in a considerable number of cases, complete temporary cure, results; and (3) that in other cases there is little or no improvement.

